# GeneXpert Technology for the diagnosis of HIV-associated tuberculosis: Is scale-up worth it?

**DOI:** 10.1515/biol-2020-0052

**Published:** 2020-06-30

**Authors:** Muhammad Saeed, Shahida Hussain, Saba Riaz, Farhan Rasheed, Maqsood Ahmad, Shagufta Iram, Mizna Arif, Tariq Hamid Rahmani, Ambereen Imran Anwar

**Affiliations:** Department of Pathology, Medical Lab Technologist-Manager Pathology & Transfusion Medicine, District Head Quarter Hospital Mandi Bahauddin, Lahore, Punjab, 50400, Pakistan; Department of Microbiology and Molecular Genetics (MMG), Punjab University Lahore, Lahore, Punjab, 54590, Pakistan; Department of Pathology, Microbiology Section, Allama Iqbal Medical College & Jinnah Hospital (AIMC&JHL) Lahore, Lahore, Punjab, 54590, Pakistan; Government College University Faisalabad, Lahore, Punjab, 38000, Pakistan; Microbiology Section, Department of Pathology, Allama Iqbal Medical College, Lahore, Punjab, 54590, Pakistan; Postgraduate Medical Institute Lahore, Lahore, Punjab, 54590, Pakistan; Department of Pathology, Ameer Iqbal Medical College Lahore, Lahore, Punjab, 54590, Pakistan; Department of Pathology, Allama Iqbal Medical College, Lahore, Punjab, 54590, Pakistan

**Keywords:** tuberculosis, HIV and TB co-infection, GeneXpert, multidrug resistance

## Abstract

Recent evaluations of the GeneXpert MTB/RIF assay for the simultaneous detection of *Mycobacterium tuberculosis* and drug resistance in less than 2 h have stimulated tremendous enthusiasm. This is the breakthrough that tuberculosis (TB) control has been waiting for. In this (retrospective review) case study, sputum samples from strongly suspected pulmonary tuberculosis patients were collected and assessed for the GeneXpert MTB/RIF assay for diagnosing TB and drug resistance in comparison with other tests, including Ziehl–Neelsen smear and Löwenstein–Jensen test. Of 3,784 cases, 5.7% (216/3,784) were human immunodeficiency virus (HIV)-positive and TB co-infected patients. In diagnosing HIV-positive and TB co-infected cases, the sensitivity and specificity of GeneXpert were 76.4% and 100%. While in HIV-negative and TB suspected cases, the sensitivity and specificity were 95.6% and 100%. This new test represents a major milestone for global TB diagnosis and care. It also represents new hope for the millions of people who are at the highest risk of TB and drug-resistant disease. GeneXpert is World Health Organization-endorsed technology representing the gold standard for TB testing despite attaining less sensitivity for HIV and TB co-infected patients as compared to HIV-negative patients.

## 1 Introduction

Synergistic tuberculosis (TB)/human immunodeficiency virus (HIV) co-infections are responsible for epidemics in several nations and have established themselves as a tremendous health challenge to these nations in terms of patient point of care and economic burden on health care organizations [1]. TB is the fundamental driver of HIV-related mortality around the world. Co-infection with these two pathogens results in an accelerated course for the two illnesses and also raises complex challenges in diagnosis and therapy [2]. Around the world, 10% of the general population who developed TB in 2016 were HIV-positive and the vast majority of these were in India, Indonesia, China, Philippines, Pakistan, South Africa and Nigeria [3].

In Eastern Mediterranean regions of the world, Pakistan is ranked the fifth highest TB endemic countries according to WHO (World Health Organization) with comparable low HIV prevalence [4,5]. From 2005 to 2011, a dramatic increase in HIV infection (10.8–27%) has been observed in the Pakistani population [5]. Among injection drug users (IDUs), the HIV infection rate is 36.7% as reported in the national surveillance program. The city-wise distribution of this positivity rate showed that Karachi has the most HIV-positive IDU cases (42.2%) followed by Lahore (30.8%) and Peshawar (20.0%). Only 7.1% cases of HIV were observed in IDUs in Quetta [6]. HIV infection is a potent risk factor for TB, and not only does HIV increase susceptibility to TB infection but it also increases the danger of prompt TB progression. The worldwide economic downturn and internal Pakistani administration issues have adversely affected the health sector particularly with respect to three key contagious diseases namely HIV/acquired immunodeficiency syndrome (AIDS), TB and TB-HIV co-infection. The overlapping clinical manifestations of TB and HIV infection result in missed, late and poor diagnosis. In HIV-positive patients, the diagnosis of pulmonary tuberculosis (PTB) is a challenging task due to the paucibacillary nature of the infection, which greatly reduces the effectiveness of smear microscopy techniques [7]. Consequently, the incidence of TB in smear-negative HIV individual cases is expected to be high [8]. The poor sensitivity of routine tests necessitates newer more sensitive and specific diagnostic approaches that are easy to implement in remote and resource constrained settings. In 2013, the WHO Global TB Program instigated a joint effort in an attempt to control the TB/HIV epidemic while minimizing costs [9].

The automated GeneXpert technology (GX-T) DNA test represents a milestone in the field of TB diagnosis and infection control [7]. To date, very few studies have investigated the performance of this promising technology in HIV-infected cases from Pakistan. Therefore, the current study was planned to determine the incidence of TB and HIV co-infection among TB patients with multidrug resistance (MDR) status, registered in the MDR Clinic in Jinnah Hospital, Lahore, assisted by the global fund fight against AIDS and TB by WHO. In this study, we also evaluate the validity of GX-T for the detection of mycobacterium tuberculosis (MTB) among (HIV positive and negative) the suspected TB cases. The findings of this project will serve to improve community health by speeding up initial diagnosis and ensuring well-timed treatment for individuals living with HIV and TB in Pakistan.

## 2 Materials and methods

### 2.1 Study setting and design

The institutional-based descriptive study was conducted at the Mycobacteriology section of the Pathology department, Allama Iqbal Medical College, Lahore, Pakistan. The Mycobacteriology section of this Pathology department is one of the largest TB referral centers in Punjab, Pakistan, where a number of samples were collected and investigated. This (retrospective review) case study was conducted during the period of 5 years from 2011 to 2015.

### 2.2 Sample size determination

The single population proportion formula of sample size (*N* = *z* ^2^ *p*(1 − *p*)/*w* ^2^) was used for the determination of sample size, where *N* is the number of suspected PTB patients; *Z* is the normal standard distribution value at 95% C; *P* is the prevalence of PTB infection = 9.9% (24); and *W* is the margin of error taken as 5%. Accordingly, a total of 3,784 sputum samples were collected from the strongly suspected cases of PTB.

### 2.3 Specimen collection and inclusion and exclusion criteria

A total of 3,784 (HIV negative = 3,568, HIV positive = 216) sputum samples were received from the strongly suspected PTB cases attending the Pulmonology department, Outdoor Patient Department, MDR clinic. The total 3,784 specimens include 216 strongly suspected TB cases with HIV positive status referred from the Punjab AIDS control program (PACP) clinic of Jinnah hospital Lahore, Pakistan. While the remaining 3,586 cases were HIV negative as assessed by immunochromatographic test screening. Patients from the PACP clinic were confirmed HIV-positive patients (PCR quantitative) and were referred for flow cytometry and GeneXpert assay in the Pathology department. Patients suspected of extrapulmonary TB infection and patients on ART were excluded from the study.


**Informed consent:** Informed consent has been obtained from all individuals included in this study.
**Ethical approval:** The research related to human use has been complied with all the relevant national regulations, institutional policies and in accordance with the tenets of the Helsinki Declaration and has been approved by the Ethical Review Board (ERB) Committee organized by Allama Iqbal Medical College, Lahore, Pakistan with reference number 40th/ERB, dated 12 August 2017.

### 2.4 Sample processing

Every sample was processed by performing Ziehl–Neelsen (ZN) smear and Löwenstein–Jensen (LJ) culture according to the standard recommendations [7]. The WHO-endorsed GeneXpert MTB/RIF assays were performed according to the manufacturer’s instructions [7]. It detects the genotype for isoniazid (INH) and rifampicin (RIF) resistance by using PCR and hybridization of bacterial DNA. In procedure, reagent was added in the sputum at the ratio of 2:1 and left it for 10 min at room temperature. It was agitated again. After 5 min, the material was transferred to the test cartridge. Then, it was placed in the GeneXpert machine, which performed whole process automatically. Electronic results were obtained for evaluation [10].

In the ZN smear test, smear was stained by the ZN staining method. Acid fast bacilli (AFB) in sputum appeared as red-colored AFB. The LJ medium was used to culture the MTB colonies.

### 2.5 Data statistical evaluation

The study population data were analyzed by using version 21 of SPSS software. The descriptive analysis was done to determine the demographic characteristic and the current prevalence of TB or TB/HIV co-infection. A series of bivariate analyses were used to determine the association between TB and TB/HIV co-infection with demographic characteristic. Data in Tables 1 and 2 are categorized into variables. Therefore, Chi-square test is used to describe the relationships between these variables.

**Table 1:** Gender and age group-based distribution of study groups (*n* = 3,784)

Age group	HIV negative				HIV positive			
	Male	Females	Transgender	Total	Male	Females	Transgender	Total
<30	375	235	47	657	49	22	11	82
30–60	967	559	63	1,589	80	18	8	106
>60	833	464	25	1,322	23	4	1	28
Total	2,175	1,258	135	3,568	152	44	20	216
*p* value	*X* ^2^ = 34.974, *p* = 0.000				*X* ^2^ = 7.855, *p* = 0.096			

**Table 2:** Gender and age group-based frequency distribution, stratified by HIV status

Age group	HIV negative TB positive				HIV + TB co-infected			
	Male	Females	Transgender	Total	Male	Females	Transgender	Total
<30	134	65	0	199	5	4	3	12
30–60	230	192	5	427	12	2	1	15
>60	448	208	2	658	6	1	0	7
Total	812	465	7	1,284	23	7	4	34
Statistics	*X* ^2^ = 28.645, *p* value = 0.000				*X* ^2^ = 6.280, *p* value = 0.179			

The Chi-square test was used to determine the sensitivity, specificity, positive predictive value (PPP) and negative predictive value (NPV) of different diagnostic tools used in the study. *P*-values of <0.05 were considered statistically significant.

## 3 Results

Of 3,784 TB suspected cases, the proportion of males, females and trans-genders were 61.4% (*n* = 2,327), 34.4% (*n* = 1,302) and 4.0% (*n* = 155), respectively, while the mean age was 38.0 + 10.0 years. The prevalence of HIV observed in this study was 5.7% (216/3,784) (Table 1). Of 216 HIV-positive TB suspected cases, 15.7% (*n* = 34) were HIV and TB co-infected, of which males, females and trans-genders were 67.6% (*n* = 23), 20.5% (*n* = 7) and 11.7% (*n* = 4), respectively (Table 2). It was observed that the sensitivity and specificity of ZN smear for HIV-negative patients was 64.7% and 100%, while it was markedly reduced for HIV and TB co-infected patients, which were 23.5% and 100%, respectively. GeneXpert showed sensitivity and specificity of 76.4% and 100% in HIV and TB co-infected patients. While in HIV-negative TB suspected cases the sensitivity and specificity were 95.6% and 100%, respectively. Overall, sensitivity, specificity, PPV and NPV of 94.5%, 100%, 100% and 97.1%, respectively, were seen for total samples. It was noted that the sensitivity of GeneXpert for smear-negative sputum samples of HIV-negative TB suspects was 85.8% and specificity 100%. GeneXpert showed remarkable and noticeable sensitivity and specificity of 69.2% and 100% in smear-negative sputum samples of TB and HIV co-infected patients (Table 3).

**Table 3:** Validity of different modalities stratified by HIV status

Patientsgroups	Techniques		LJ culture		Total	Sensitivity (%)	Specificity (%)	PPV(%)	NPV(%)	Statistics
			+ve	−ve						
HIV −ve	Zn smear	+ve	832	0	832	64.7	100	100	83.4	*X* ^2^ = 1930.26 *p* = 0.000
		−ve	452	2,284	2,736					
		Total	1,284	2,284	3,568					
HIV +ve	Zn smear	+ve	8	0	8	23.5	100	100	87.0	*X* ^2^ = 44.471 *p* = 0.000
		−ve	26	182	208					
		Total	34	182	216					
Total	Total	+ve	840	0	840	63.7	100	100	83.7	*X* ^2^ = 2020.088 *p* = 0.000
		−ve	478	2,466	2,944					
		Total	1,318	2,466	3,784					
HIV −ve	GeneXpert	+ve	1,220	0	1,220	95.0	100	100	97.2	*X* ^2^ = 3276.54 *p* = 0.000
		−ve	64	2,284	2,348					
		Total	1,284	2,284	3,568					
HIV +ve	GeneXpert	+ve	26	0	26	76.4	100	100	95.7	*X* ^2^ = 158.222 *p* = 0.000
		−ve	8	182	190					
		Total	34	182	216					
Total	GeneXpert	+ve	1,246	0	1,246	94.5	100	100	97.1	*X* ^2^ = 13475.803 *p* = 0.000
		−ve	72	2,466	2,538					
		Total	1,318	2,466	3,784					
HIV −ve smear negative	GeneXpert	+ve	388	0	388	85.8	100	100	97.2	*X* ^2^ = 2456.979 *P* = 0.000
		−ve	64	2,284	2,348					
		Total	452	2,284	2,736					
HIV +ve smear negative	GeneXpert	+ve	18	0	18	69.2	100	100	95.7	*X* ^2^ = 137.937 *P* = 0.000
		−ve	8	182	190					
		Total	26	182	208					
Total smear negative	GeneXpert	+ve	406	0	406	84.9	100	100	97.1	*X* ^2^ = 2429.615 *P* = 0.000
		−ve	72	2,466	2,538					
		Total	478	2,466	2,944					

**Figure 1:**
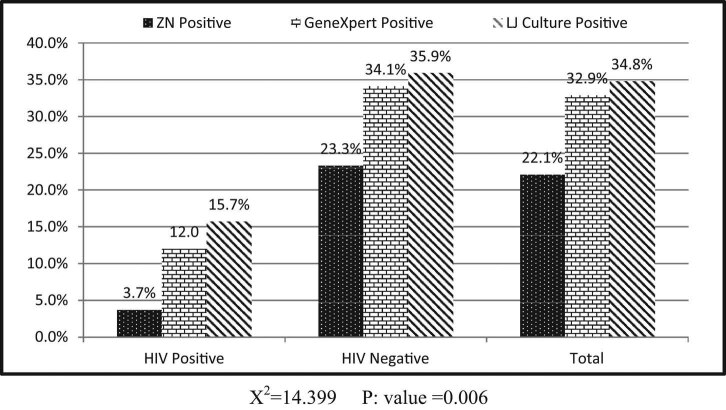
Detection of MTB by different techniques stratified by HIV status.

**Figure 2:**
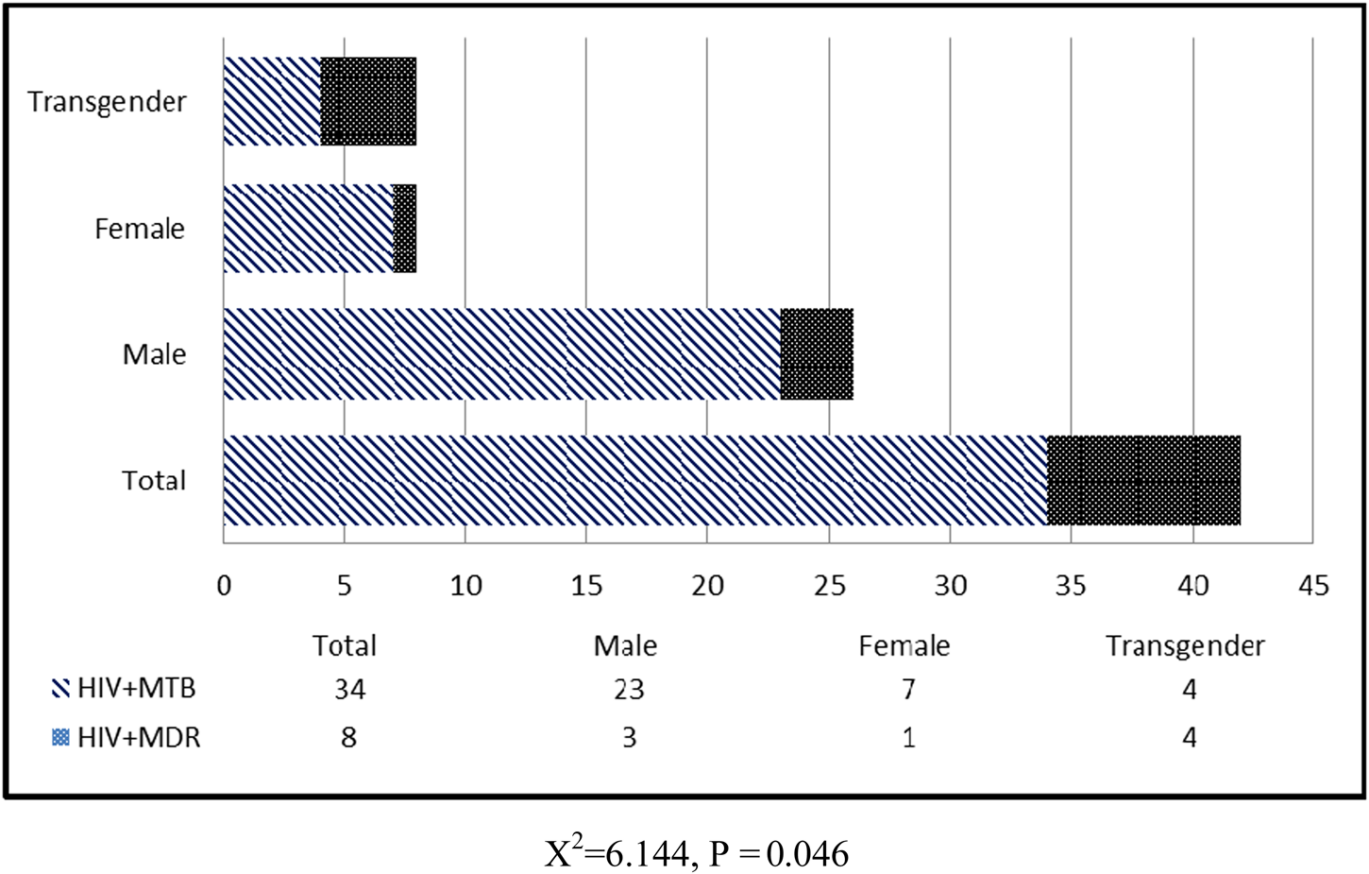
Community-wise frequency distribution of MDR cases detected by GeneXpert.

**Figure 3:**
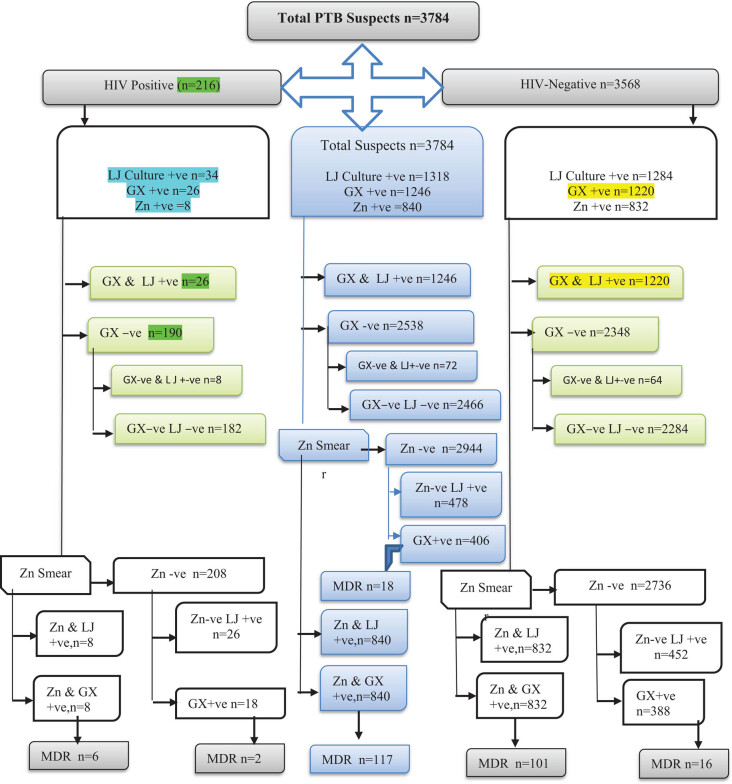
Flow chart of sample processing and summary of results.

The detection rates of conventional microbiological techniques (ZN and LJ culture) and GeneXpert are depicted in Figure 1. The maximum cases were detected by LJ culture (*n* = 1,318), followed by the GeneXpert (MTB/RIF) assay (*n* = 1,246) and ZN smear (*n* = 840), respectively. Among 216 HIV and TB co-infected patients, 30.7% (*n* = 8/26) were MDR. Out of 1,220 HIV-negative TB-positive cases detected by GeneXpert, 9.5% (*n* = 117) were MDR (Figure 2). The complete scheme of the project is depicted in Figure 3.

## 4 Discussion

Pakistan is a country with a population of 176 million, located in the middle of Asia and shares its boundaries with South Asia, Central Asia and the Middle East. Pakistan ranks fifth in the world for new TB cases per year behind China, India, South Africa and Indonesia, which demonstrates the massive challenges with inadequate resources and conflicting priorities [11]. Pakistan is a high TB but low HIV burden country; on the other hand, the increased risk among certain subgroups within the population has been noted since 2005 [11].

In our study, we report overall HIV incidence of 5.7% (216/3,784) in TB suspected cases attending the Jinnah Hospital, Lahore, Pakistan. The TB infectivity rate can vary from region to region depending upon the HIV status, and previous studies have reported HIV positivity ranging from 3.1% to 70.0% in different geographical regions of the world [12]. The TB-HIV co-infection rate observed in our study was 15.7% (34/216). Previous studies have reported lower HIV–TB co-infection rates. A similar study conducted on the Pakistani population by Hussain et al., in Sindh, Pakistan, reported 0.34% HIV–TB co-infections [13]. A study done by Channa and a study done by Arrora reported HIV–TB co-infection rates of 1.34% and 2.5%, respectively, which is significantly lower than our findings [14,15], but Scott et al. reported a higher rate of 38.5% [16]. The variation in study population and area under investigation has an influence on the prevalence of infectious diseases, as HIV infectivity rates are reported in the range of 1.7% to 76% in a multi-country study conducted in different geographical cities [17]. This alarming emerging problem will be a great challenge for the control of TB and HIV.

This study was planned to assess the validity of WHO-endorsed GX-T in diagnosing TB in HIV-negative/positive TB suspected cases. The GeneXpert showed a sensitivity and a specificity of 95.0% and 100% for the detection of MTB in TB suspected cases, and these findings are in agreement with previous studies [7,12,17]. GeneXpert showed a good sensitivity and a specificity of 69.2% and 100%, respectively, for detecting MTB in smear-negative, LJ-positive, HIV-positive patients. Carriquiry et al. reported that among 131 HIV-positive patients, the sensitivity and specificity of the GeneXpert assay for the detection of MTB were 97.8% and 97.7%, respectively [18]. Scott et al. reported the sensitivity of the GeneXpert assay as 61.0% in smear-negative-culture-positive samples [16]. Al-Darraji et al. reported a sensitivity and a specificity of 53.3% and 100% in HIV-infected prisoners by GeneXpert [19]. These findings together with our findings build a strong case for the utilization of the GeneXpert assay in the diagnosis of TB in HIV-positive patients due to its rapid results and remarkably improved sensitivity as compared to conventional AFB smear microscopy techniques such as ZN.

Diagnosis of active TB by a sputum-based assay with a sensitivity of 85% and a specificity of 97% has the potential to save >400,000 lives per year [20]. The emergence of drug resistance is a very serious problem for TB control authorities. MTB bacteria which develop the resistance against antimicrobial drugs, particularly, INH and RIF (two most powerful anti-TB drugs), have become MDR-TB [21].

The GeneXpert MTB/RIF assay is considered a good indicator for MDR-TB, and it detects mutations in the rpoB gene which occur in 95–99% of the RIF-resistant isolates [22,23]. In this study, the GeneXpert MTB/RIF assay identified 125 patients composed of 8 HIV and TB co-infected and 117 HIV-negative MDR-TB cases, it greatly reduced the time of detection (2 h) as compared with conventional culture-based susceptibility testing (40 days). By significantly reducing diagnosis time, the GeneXpert assay has the potential to substantially reduce the risk of nosocomial transmission of MDR-TB and improve the prognosis of affected individuals. HIV-associated MDR-TB patients carry a very high mortality risk and can cause nosocomial outbreaks in HIV care and treatment centers posing a grave threat to patients accessing these services [24,25]. The literature shows that many HIV-associated MDR-TB patients die before a diagnosis can be made [24,25]. Prompt initiation of therapy of HIV–TB patients could improve individual prognosis and reduce the global TB disease problem to the benefit of all [26,27].

The GeneXpert assay is recommended in the diagnosis of both pulmonary and extrapulmonary TB among HIV-positive/negative patients of every age, and it is a very rapid screening test for MTB and MDR-TB. Its uniqueness to detect MTB and MDR-TB in a single process at the same time is a major advantage. The present model in Pakistan is only to detect MTB along with RIF resistance, but WHO has announced its latest model for the near future employing assays such as GeneXpert Prime and GeneXpert Ultra with many additional diagnostic capabilities for drug-resistant TB [28].

The lack of a reliable and prompt diagnostic test for the identification of smear-negative active TB cases in adults and especially children represents a serious cross infection risk. On the basis of the clinical picture alone patients are frequently started on potentially harmful anti-TB treatment incorrectly. Equally various other opportunistic infections associated with HIV patients mimic the clinical appearance of TB which can result in unnecessary drug administration, risking adverse drug responses, medication interactions and development of resistance [29]. According to the WHO guidelines every TB-positive patient should be screened for HIV. Only 51% of the notified TB cases had documented HIV test results around the globe in 2014, which is only a small improvement from 49% in 2013. Unfortunately, Pakistan is ranked in the low to middle-income countries, but it is crucially important that funding gaps are closed to make every effort to develop new tools to combat this national and global menace [6,11,30].

## 5 Conclusions

GeneXpert is the WHO-endorsed technology that is proven to be highly sensitive and specific for the diagnosis of HIV–TB co-infected patients. GeneXpert assay sensitivity is less in HIV-positive patients as compared to HIV-negative patients. But its sensitivity is far more than ZN microscopy in any case. “This is because people in later stages of HIV infection and with compromised immune systems often release fewer organisms into their sputum.” HIV causes alterations in the host immune system response to MTB and as a result cavitation and transfer of bacilli into respiratory secretions are evidently reduced (https://www.who.int/tb/challenges/hiv/Xpert_TBHIV_Information_Note_final.pdf). Therefore, GeneXpert attained less sensitivity for HIV and TB co-infected patients as compared to HIV-negative patients; however, it seems to be close to the gold standard for TB testing.

## Abbreviations

AFB: acid fast bacilli

AIDS: acquired immunodeficiency syndrome

ERB: Ethical Review Board

GX-T: GeneXpert technologyd

HIV: human immunodeficiency virus

IDU: injection drug users

LJ: Löwenstein–Jensen

MDR: multidrug resistance

MTB: mycobacterium tuberculosis

NPV: negative predictive value

PPV: positive predictive value

PTB: pulmonary tuberculosis

TB: tuberculosis

WHO: World Health Organization

ZN: Ziehl–Neelsen
